# Implementation of the case management and interdisciplinary approach in an innovative integrated services delivery in France

**Published:** 2012-09-04

**Authors:** Matthieu de Stampa, Dominique Somme, Catherine Périsset, Olivier Dupont, Nadia Arnaout, Joël Ankri, Olivier Saint-Jean

**Affiliations:** Hôpital Sainte Perrine, and Expert for the National Project Team, Ministère du Travail de l’Emploi et de la Santé, Paris; Hôpital Pompidou, and Expert for the National Project Team, Ministère du Travail de l’Emploi et de la Santé, Paris; Expert Project Manager for the National Project Team, Caisse Nationale de Solidarité pour l’Autonomie, Paris; Project Executive Manager for the National Project Team, Caisse Nationale de Solidarité pour l’Autonomie, Paris; Administrative Manager for the National Project Team, Caisse Nationale de Solidarité pour l’Autonomie, Paris; Hôpital Sainte Perrine, Versailles University; Hôpital Pompidou, and René Descartes University, Paris

**Keywords:** case management, interdisciplinary practices, integrated services delivery

## Abstract

**Purpose:**

The 2008–2012 French Alzheimer plan is marked by the experimental implementation of “Homes for Integration and Autonomy for Alzheimer patients” with implementation of intensive case management process for people in complex situation. In 17 experimental sites, teams of case managers (CMs) have been implemented with a total of 67 case managers for performing an intensive case management.

**Methods:**

We conducted a qualitative study to develop a full understanding concerning the implementation of the case management process and the links with the interdisciplinary approach. Focus groups were conducted in 2010 to explore the perceptions of case managers six months after their recruitment.

**Results:**

Interdisciplinary approach inside the case managers’ team has been rapidly constructed to resolve the complex situations, homogenize practices and help to create the new competency. Multidisciplinary and proximity team, common training and difficulties to get the good fit with professionals increased the interdisciplinary approach inside the case managers’ team. Interdisciplinary approach between case managers and clinical professionals was few implemented because of the high level of fragmentation and lack of case managers legitimacy.

**Discussion:**

Implementation of intensive case management in an innovative integrated services delivery showed a stronger interdisciplinary approach between case managers compared to with clinical professionals.

Powerpoint presentation available at http://www.integratedcare.org at congresses – San Marino – programme.

